# Transport Characteristics of Interfacial Charge in SiC Semiconductor–Epoxy Resin Packaging Materials

**DOI:** 10.3389/fchem.2022.879438

**Published:** 2022-04-26

**Authors:** Chi Chen, Jiaxing Li, Xia Wang, Kai Wu, Chuanhui Cheng, Chuang Wang, Yuwei Fu

**Affiliations:** ^1^ School of Electrical Engineering, Xi’an University of Technology, Xi’an, China; ^2^ State Key Laboratory of Electric Insulation and Power Equipment, Xi’an Jiaotong University, Xi’an, China; ^3^ Electric Power Research Institute, China Southern Power Grid, Guangzhou, China

**Keywords:** epoxy, SiC materials, interface charge, carrier transport, charge dissipation

## Abstract

The silicon carbide (SiC) wide bandgap (WBG) semiconductor power device has been widely applied for its excellent properties. However, the charge accumulated in the interface of SiC semiconductor-related insulation packaging may lead to serious material performance degradation and failure, threatening the reliability and operation life of power devices. In this research, interface charge accumulation characteristics of SiC–epoxy resin double-layered material were investigated, and space charge injection, transport, and accumulation mechanisms, as well as the related temperature effect for the SiC–epoxy resin under polarization and depolarization conditions, were studied by the pulsed electro-acoustic (PEA) technique. The results show that a charge peak appears between the SiC–epoxy resin interface, which shows the same polarity as the SiC side electrode. Charge injects from the SiC electrode, transports along with the SiC semiconductor bulk due to the high mobility, and is blocked by the interface barrier. In addition, under high temperature and high electrical stress conditions, obvious charge accumulation occurs inside the epoxy resin bulk, which was captured by the deep traps. The charge accumulation of the SiC-insulation packaging material can influence the operation of the power device and should attract more attention.

## Introduction

Recently, great progress has been made in the development of power chips and modules. High voltage, high power density devices, and modules are the significant directions (Konstantinov et al., 1997). At present, new type wide bandgap (WBG) semiconductor materials, such as silicon carbide (SiC) and gallium nitride (GaN), have gradually been widely researched because of their excellent electrical, mechanical, and thermal properties (Kong et al., 1988; Palmour et al., 1988; Suttrop et al., 1992; Liu et al., 2019). SiC WBG power devices and modules are widely used in automotive motor drive, oil gas drilling and extraction, avionics power supply, military, and other fields (Götz et al., 1993; Asada et al., 2015). To improve the power conversion efficiency and increase the power density, the size of the high-voltage power module should be small to a certain extent, while the working voltage and temperature are improved constantly, which puts forward higher requirements for the SiC and related insulation packaging materials. However, the invalidity of SiC WBG and the related packaging material is an inevitable issue that restricts the development of SiC WBG power devices in the field of high voltage and high current (Delage and Dua, 2003; Oh et al., 2015).

Space charge is the important inducement and internal mechanism of material degradation and invalidity. Under the blocking condition of SiC WBG power devices, a charge carrier can be injected and accumulated inside the SiC WBG semiconductor and insulation packaging material. In addition, chemical defects in polymer materials will introduce deep traps and become defect points that allow carriers to stay, which provides conditions for space charge accumulation, and greater energy is required for the trapped charge to escape (Teyssedre and Laurent, 2005). The charge used for transporting and trapping will accelerate the electrical aging and even breakdown of these materials, leading to the aggravation of the materials’ invalidity. The accumulation of the space charge can distort local electrical stress distribution and the discharge, aggravating the material property degradation and failure. In addition, during the operation of SiC WBG power devices, the high temperature effect caused by the device heating could lead to the charge carrier transportation and accumulation becomes more complicated. To investigate the mechanism of the invalidity of the SiC semiconductor and related insulation packaging material, it is significant to study the charge carrier transportation and accumulation in the SiC-packaging material first.

Until now, extensive research has been carried out on the invalidity of the SiC WBG semiconductor and the related packaging material (Chi et al., 2020). Published reports are mainly focused on the breakdown voltage, leakage current (Schoeck et al., 2018; Indari et al., 2019; Long et al., 2020), and the defect characteristic of SiC semiconductors (Jacobson et al., 2002; Jacobson et al., 2004; Skowronski and Ha, 2006; Peng et al., 2016). SiC material has 3 times the bandgap width of the silicon material, 10 times the critical breakdown electric field strength of the silicon material, and 3 times the thermal conductivity of the silicon material (Ren and Xu, 2020). Also, much emphasis has been placed on the partial discharge, aging, and electrical tree of semiconductors’ packaging materials (Auge et al., 2013; Locatelli et al., 2014; Sato et al., 2015). However, the research on the charge carrier transportation and accumulation in the SiC WBG semiconductor and the related insulation packaging materials is not reported yet. Also, it still lacks an understanding of space charge effect between the SiC WBG semiconductor and the related packaging material.

In this research, the charge carrier injection, transport, accumulation, and dissipation mechanisms in the SiC–epoxy resin packaging material were discussed. Interface charge behaviors were studied under polarization and depolarization conditions based on the pulsed electro-acoustic (PEA) space charge measurement technique. It reveals that the charge carrier can be injected from the electrode, transported along the SiC WBG semiconductor, and blocked by the SiC–epoxy resin interface barrier, causing an obvious accumulation of interface charge. Also, homo-charge accumulation also appears inside the epoxy resin packaging material. This research mainly focuses on the charge transport dynamic on SiC WBG semiconductors. The charge accumulation and local electrical stress distortion in SiC semiconductors and the related packaging material can influence the normal operation of the power devices and should attract more attention.

## Experimental Method

**FIGURE 1:**
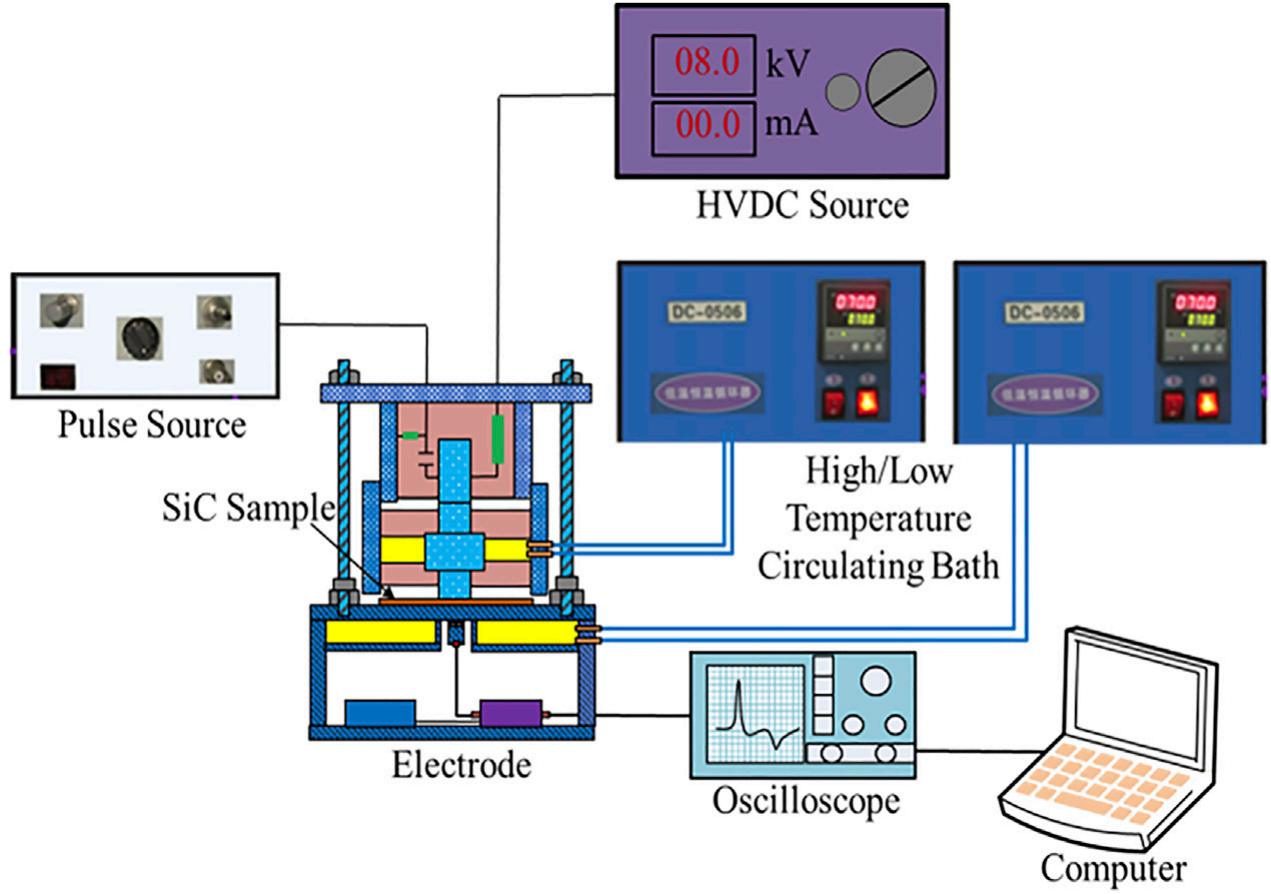
Schematic diagram of PEA space charge measuring system for the SiC–epoxy sample.

In this article, the 4H-SiC WBG semiconductor and epoxy resin insulation materials were used to study the interface charge characteristics. The thickness of these two samples was 0.3 and 0.2 mm, respectively. The micropipe density of the measured SiC crystal material is 20/cm^2^. The vanadium-doped concentration is 2.6 × 10^16^/cm^3^. This information is provided by the SiC wafer manufacturer. Also, gold electrodes were sputtered on each side of the SiC and epoxy resin materials. The space charge for the SiC–epoxy resin double-layered sample was measured with the pulsed electro-acoustic (PEA) space charge experiment method. Figure 1 shows the schematic diagram of the measuring device. During the space charge measurement, the SiC–epoxy resin double-layered plate sample was put into the space charge-measured electrode. A nanosecond pulse source was used to apply the high-frequency nanosecond pulse signal, while the HVDC source was used to apply the high-voltage signal. In this research, the SiC semiconductor side was set to the anode electrode, while the epoxy resin side was the cathode electrode. Also, through the high temperature circulating bath, the temperature condition for both the higher and lower surfaces of the double-layered sample could be set to a fixed value. The data acquisition system of measurement equipment mainly consists of an amplifier, oscilloscope, and computer and was used to process the measured PEA signal furthermore.

In this research, the space charge was measured under 10, 15, and 20 kV/mm DC electrical stresses, and 30, 50, and 70°C temperature conditions, respectively. The polarization time was set at 20 min, while the depolarization time was set at 10 min. After the measurement, the space charge profiles were calibrated and recovered based on the wave propagation law in the two samples. To improve the accuracy of charge data processing, first, the background clutter was collected and subtracted by each measured space charge waveform.

## Results and Discussion

**FIGURE 2:**
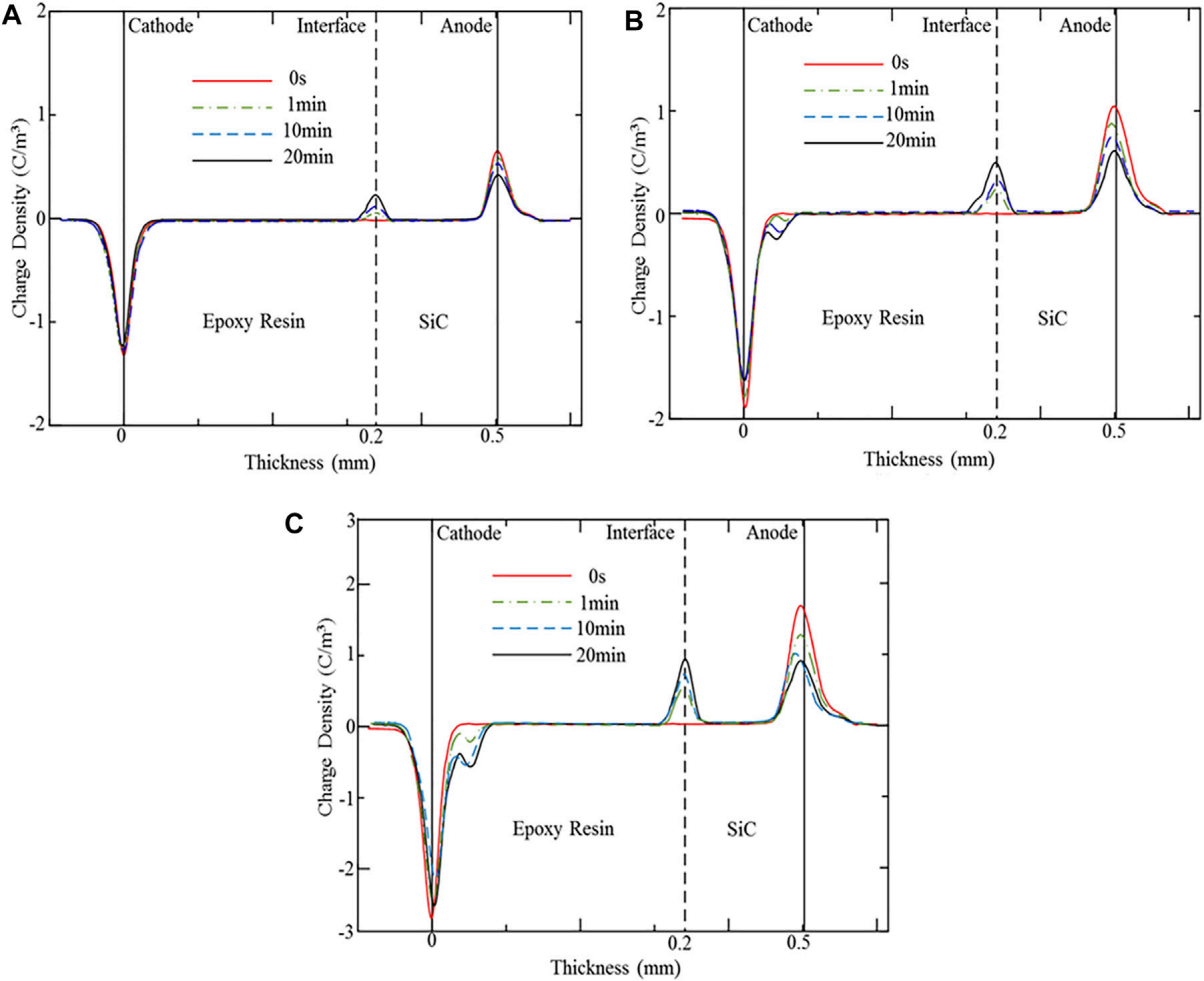
Space charge profiles for the SiC–epoxy resin double-layered sample with different polarization time. **(A)** Space charge distribution at 10 kV/mm. **(B)** Space charge distribution at 15 kV/mm. **(C)** Space charge distribution at 20 kV/mm.

Space charges for the SiC–epoxy resin double-layered sample are presented in Figure 2. An obvious interface charge peak appears in the interface of the SiC–epoxy resin double-layered sample, which shows the same polarity with the SiC electrode (anode) charge peak. Also, the interface charge peak increases with the polarization time, while the SiC electrode charge peak decreases. Also, almost no charge appears inside the epoxy resin bulk under 10 kV/mm, while obvious homo-charge accumulates near the epoxy resin electrode (cathode) under 15 and 20 kV/mm. Therefore, the accumulation of the interface charge is mainly originated from the charge carrier injected from the SiC WBG electrode; another way of the source of the interface charge is impurity ionization. After injection from this electrode, the charge carrier can transport along with the SiC bulk rapidly because of the high mobility of SiC WBG semiconductor material. Also, the migrated carrier would be blocked by the SiC–epoxy resin interface due to the interface barrier, which is caused by the nonideal interface contact and additional interface states. A small part of the charge carrier may pass through the interface, but it cannot be indicated by the space charge profiles because of the limit of the resolution of the PEA measurement technique.

**FIGURE 3:**
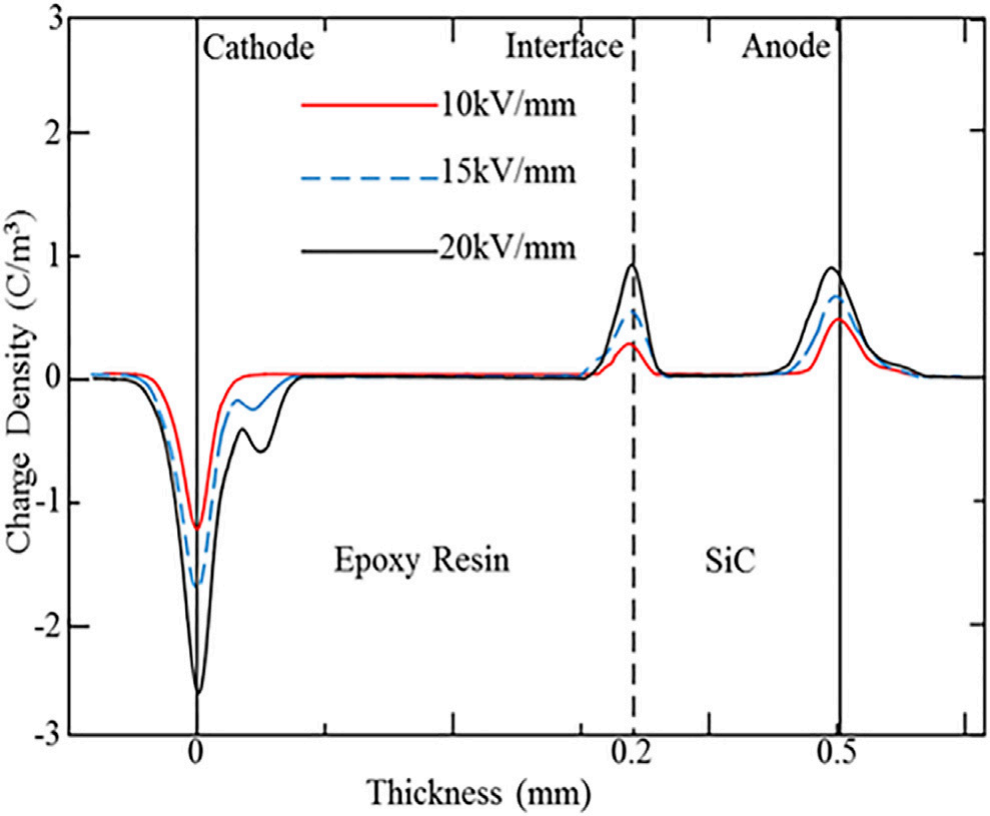
Space charge profiles for the SiC–epoxy resin double-layered sample under different DC electrical stresses.

In addition, due to the higher injection barrier of the epoxy resin electrode interface, the charge carrier cannot be injected into the epoxy resin under 10 kV/mm. Under 15 and 20 kV/mm DC stress, carriers can be injected from the epoxy electrode and captured by deep traps near the electrode interface. Due to the high energy level of deep traps, the trapped charges are not easy to fall off, which is equivalent to reducing the overall mobility of the carriers, causing homo-charge accumulation (Montanari et al., 2001; Mazzanti et al., 2003). Almost 20 min later, after the application of DC electrical stress, space charge profiles of the SiC–epoxy resin double-layered sample reach an equilibrium state. Also, to analyze the influence of the applied DC electrical stress, the space charge profiles of the SiC–epoxy resin double-layered sample under a steady-state are collected together, which is presented in Figure 3.

It could be concluded that both the interface charge and the homo-charge inside the epoxy resin increase with the applied DC electrical stress. The amplitude of the three interface charge peaks is 0.4, 0.6, and 1.0 C/m^3^, while the homo-charge amplitudes are 0, 0.3, and 0.7 C/m^3^. Meanwhile, the homo-charge near the epoxy resin electrode extends into the sample bulk. Therefore, the injection rate of the charge carrier from the two electrodes increases with the applied DC electrical stress. With the aggravation of charge injection from the epoxy resin electrode, the partial carrier can transport inside the bulk, causing an extension of the homo-charge.

**FIGURE 4:**
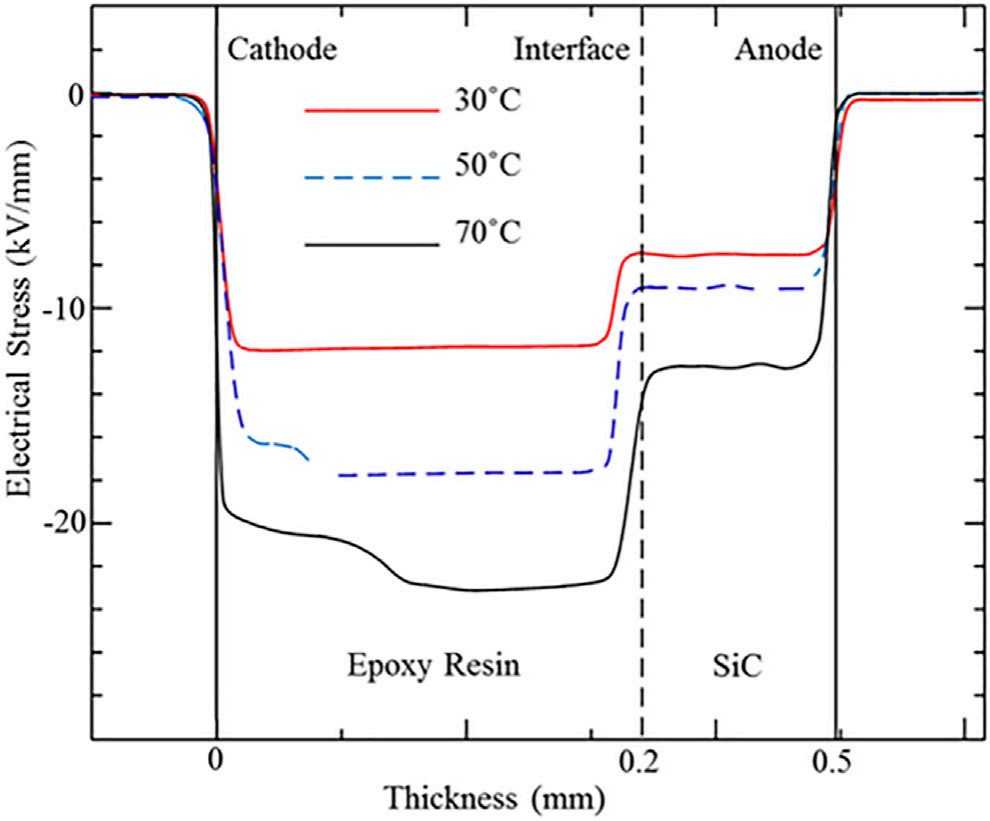
Electrical field distribution for the SiC–epoxy resin double-layered sample under different applied DC electrical stresses.

The related local electrical field distribution of the SiC–epoxy resin double-layered sample is also calculated from the space charge profiles through the Poisson equation, as shown in Figure 4. It can be seen from Figure 4 that the accumulation of the interface charge distorts the local electric field distribution inside the SiC–epoxy resin double-layered sample. The electrical field inside the SiC WBG semiconductor decreases, while it increases inside the epoxy resin. This is mainly because the interface charge is injected from the SiC electrode and shows the same polarity as the electrode. The injection rate of the two electrodes is also influenced by the distortion of the local electrical field. In addition, the local electric field distribution near the epoxy resin electrode decreases due to the accumulation of the homo-charge under 15 and 20 kV/mm. Also, it could be seen from Figure 4 that the largest negative electrical field appears inside the epoxy resin, while the most serious distorted electrical field exists in the SiC–epoxy resin interface. Also, the local electrical field at the interface from the SiC WBG semiconductor and the epoxy resin changes rapidly.

**FIGURE 5:**
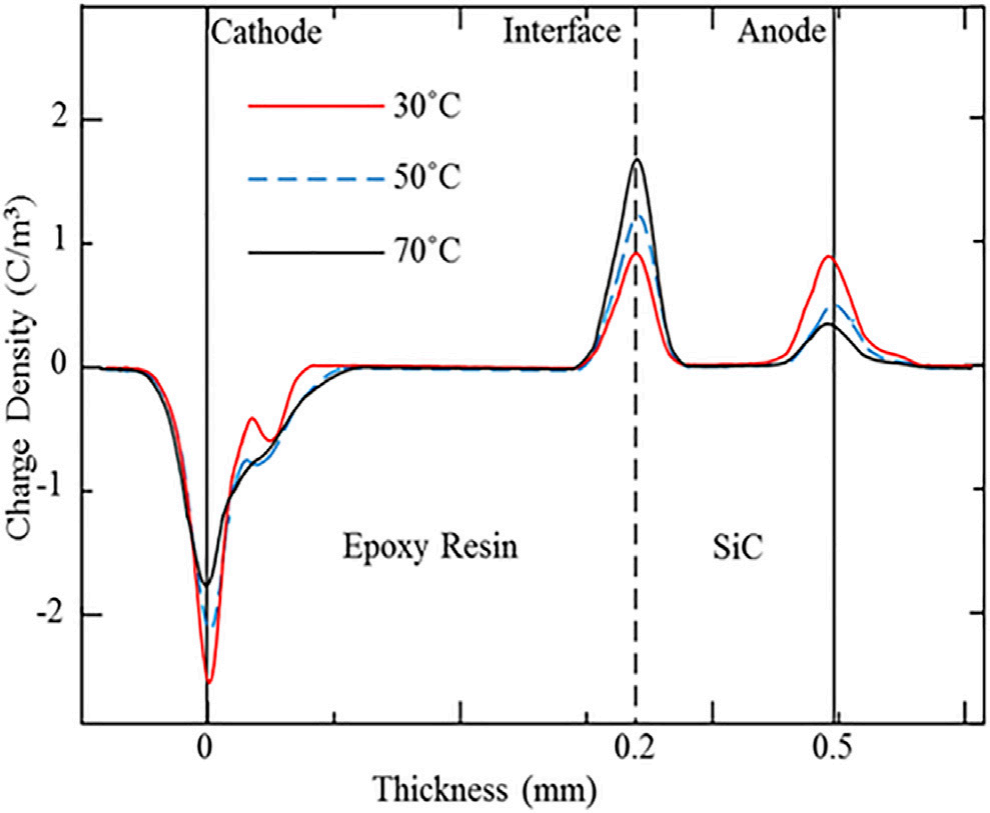
Space charge profiles for the SiC–epoxy resin double-layered sample under different temperature conditions.

**FIGURE 6:**
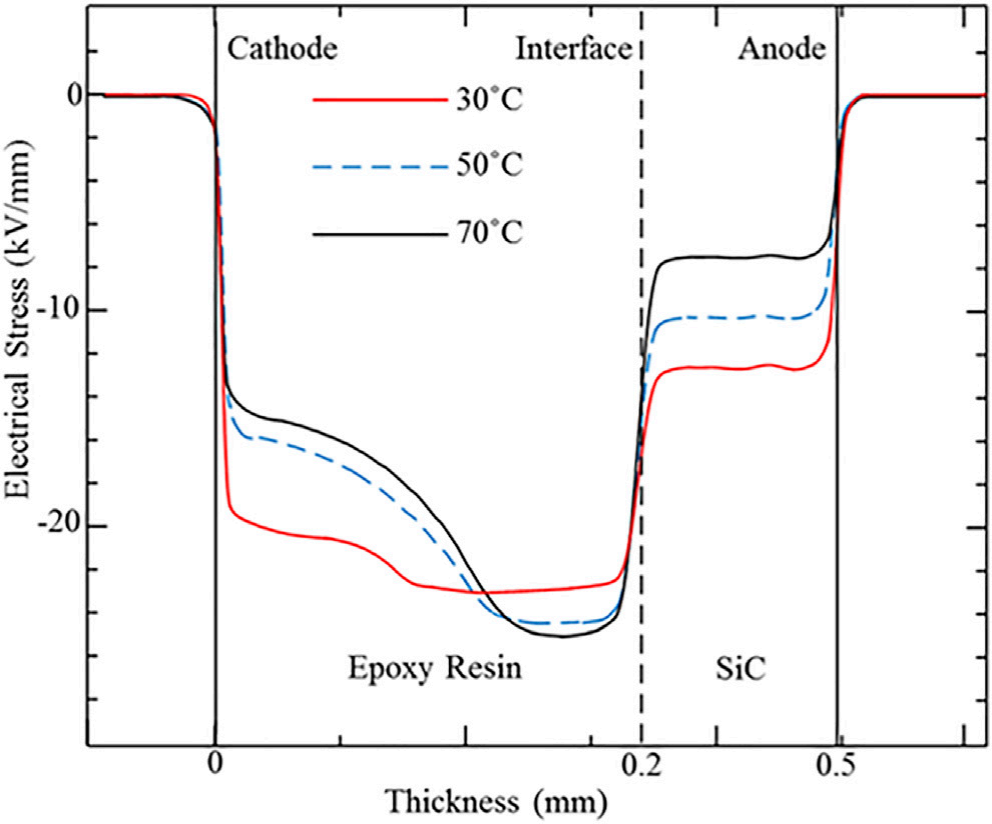
Electrical field distributions for the SiC–epoxy resin double-layered sample under different temperature conditions.

To study the influence of the high temperature effect caused by the fever of the power devices, the charge carrier transportation and accumulation under the high temperature conditions (30, 50, and 70°C) were also measured and analyzed in this research. Figures 5, 6 present the space charge and electric field distribution profiles of the SiC–epoxy resin under different temperature conditions. As Figure 5 shows, the interface charge peak increases, while the two electrode peaks decrease with the temperature. Therefore, the high temperature effect can aggravate the injection rate of both the epoxy resin insulation material and SiC WBG semiconductor electrodes, so both the accumulated homo-charge and the interface charge increase with the temperature. In addition, the migration rate of the charge carrier in the epoxy resin also improves with the temperature, and these two factors cause the accumulated charge to extend into the epoxy resin furthermore. From Figure 6, the largest electrical stress in the epoxy resin and the distortion of the interface electrical field also increase with the temperature. Therefore, during the operation of SiC WBG semiconductor power devices, the interface position between the SiC and the related packaging insulation material should be paid more attention to as the electric field concentration and distortion.

It could be deduced that the charge carrier injection from the SiC electrode can improve the interface charge, while its injection from the epoxy resin electrode aggravates the extension of the homo-charge into the sample and toward the interface. As the injection and migration of charge carrier increase with the applied DC electrical stress and temperature condition, the charge carrier injection from the epoxy resin electrode may transport and accumulate near the interface at high temperature and high DC electrical stress conductions, resulting in a hetero-charge distribution in the SiC–epoxy resin interface. The carrier transport and accumulation under such a higher temperature will be studied in our following research.

**FIGURE 7:**
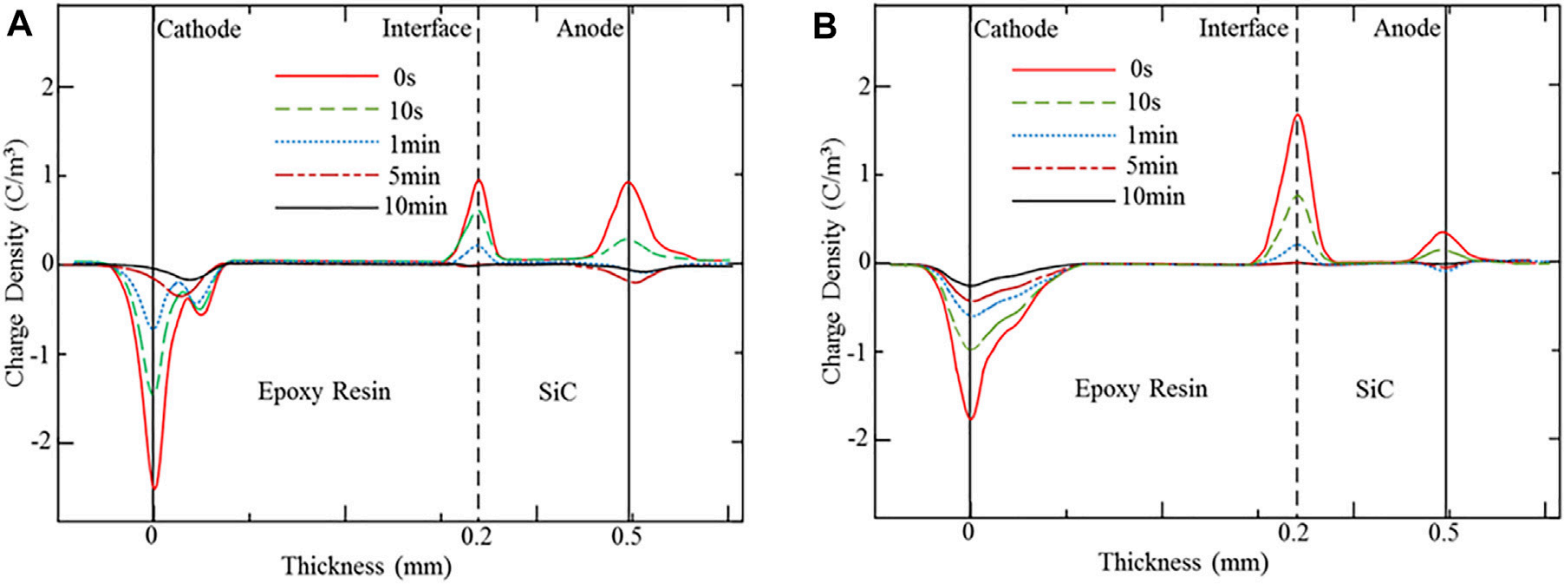
Depolarization charge profiles for the SiC–epoxy resin double-layered sample under different temperature conditions. **(A)** Depolarized charge distribution at 30°C. **(B)** Depolarized charge distribution at 70°C.

To investigate the depolarization charge characteristic and residual charge behavior of the SiC–epoxy resin material, the short circuit space charge profiles are measured and analyzed. At 20 kV/mm, the short-circuit charge profile at 30°C and 70°C are shown in Figures 7A and B respectively.

It can be seen that the SiC electrode charge peak dissipates to the noise level rapidly, just takes almost 10 , whereas the interface charge peak dissipates a little slowly. Also, the charge peak at the epoxy resin electrode dissipates relatively slow, that is, 10 min later, some residual charge still exists. Because of the higher mobility of the SiC WBG semiconductor than that of the epoxy resin, the interface charge mainly dissipates through the SiC electrode rapidly after depolarization, and the existence of the interface barrier limits the dissipation rate. Also, at the epoxy resin electrode, the capacitive charge can dissipate relatively quickly. However, the charge carrier captured by the deep traps cannot be extracted easily, and most of them stay in the epoxy resin as the residual charge. Also, under the higher temperature, the residual charge extends a little far away from the epoxy resin electrode, as shown in Figure 7B.

## Conclusion

In this article, the carrier transportation and accumulation in the SiC–epoxy resin double-layered sample were measured and analyzed with the PEA space charge experimental technique, and the interface charge behaviors were investigated. The results showed that an obvious interface charge peak appears in the SiC–epoxy resin interface, which shows the same polarity with the SiC electrode charge peak. Also, the homo-charge appears near the epoxy resin electrode and extends toward the sample bulk. These interface charges and homo-charge increase with the temperature and DC electrical stress. The accumulation of the space charge causes an obvious distortion of the local electrical field distribution, resulting in the electrical field changing rapidly at the interface. Also, after depolarization, the SiC semiconductor charge peak dissipates rapidly, while the dissipation of the interface charge peak shows little slowness. Also, several minutes later, a residual charge still exists inside the epoxy resin bulk.

This interface charge peak between the SiC WBG semiconductor and the epoxy resin is originated from the injection of the charge carrier from the SiC electrode. After injection, these carriers transport along the SiC bulk rapidly due to the high mobility of SiC WBG semiconductors. Then, these charge carriers are blocked and accumulated at the interface due to the interface barrier. Also, with the increase of the injection rate at high temperature and high electrical stress, the interface charge peak could increase. In addition, the accumulated homo-charge in the epoxy resin was caused by the injection of electrodes and trapping by the deep traps. After depolarization, the interface charge dissipation is limited by the interface barrier, so it could not dissipate rapidly, while the residual charge captured by the deep trap stays inside the epoxy resin for a relatively long time.

## Data Availability

The original contributions presented in the study are included in the article/Supplementary Material. Further inquiries can be directed to the corresponding author.
